# Changes in Overall Participation Profile of Youth with Physical Disabilities Following the PREP Intervention

**DOI:** 10.3390/ijerph17113990

**Published:** 2020-06-04

**Authors:** Colin Hoehne, Brittany Baranski, Louiza Benmohammed, Liam Bienstock, Nathan Menezes, Noah Margolese, Dana Anaby

**Affiliations:** 1 Health Sciences Centre, Winnipeg, MB R3A 1R9, Canada; choehne@hsc.mb.ca; 2 School of Physical & Occupational Therapy, McGill University, Montreal, QC H3G 1Y5, Canada; brittany.baranski@mail.mcgill.ca (B.B.); liam.bienstock@mail.mcgill.ca (L.B.); nathan.menezes@mail.mcgill.ca (N.M.); noah.margolese@mail.mcgill.ca (N.M.); 3 CIUSSS du Nord-de-l’Île-de-Montréal, Montreal, QC H4K 1B3, Canada; louiza.benmohammed@mail.mcgill.ca; 4 Centre de Recherche Interdisciplinaire en Réadaptation de Montréal Métropolitain (CRIR), Montreal, QC H3S1M9, Canada

**Keywords:** social participation, adolescence, intervention

## Abstract

The Pathways and Resources for Engagement and Participation (PREP), an environmental-based intervention, is effective in improving the participation of youth with disabilities in specific targeted activities; however, its potential impact on overall participation beyond these activities is unknown. This study examined the differences in participation levels and environmental barriers and supports following the 12-week PREP intervention. Existing data on participation patterns and environmental barriers and supports, measured by the Participation and Environment Measure for Children and Youth, pre-and post-PREP intervention, were statistically analyzed across 20 youth aged 12 to 18 (mean = 14.4, standard deviation (SD) = 1.82) with physical disabilities in three settings: home, school and community. Effect sizes were calculated using Cohen’s *d*. Following PREP, youth participated significantly less often at home (*d* = 2.21; 95% Confidence Interval (CI) [1.79, 2.96]), more often (*d* = 0.57; 95% CI [−0.79, −0.14]) and in more diverse activities (*d* = 0.51; 95% CI [−1.99, −0.51]) in the community. At school, significantly greater participation was observed in special school roles (t = −2.46. *p* = 0.024). Involvement and desire for change remained relatively stable across all settings. A substantial increase in community environmental supports was observed (*d* = 0.67), with significantly more parents reporting availability of, and access to information as a support (χ^2^ = 4.28, *p* = 0.038). Findings lend further support to the effectiveness of environmental-based interventions, involving real-life experiences.

## 1. Introduction

Participation, defined as involvement in life situations [1], involves being with others [2], and is critical to the development of physical, emotional and social well-being in youth with and without disabilities [3,4]. Through participation, youth can develop a sense of self, feelings of success and connectedness to their community [5]. Participation of youth is of particular importance not only because their participation levels decrease as they enter adolescence [6] but also because they face a challenging transitional phase to adulthood [7]. Indeed, youth living with disabilities report lower participation levels than those without disabilities at home, school and in the community [8,9]. Specifically, they experience lower participation frequency, lower involvement levels and poorer satisfaction with their participation profile [10].

Some of the key factors that determine the participation profile of youth with disabilities lie within their environment [11]. Recent scoping reviews reveal a range of common environmental barriers and supports which can affect participation [12,13,14]. Examples of barriers include physical inaccessibility, unsupportive attitudes of others and lack of knowledge about ways to adapt activities and equipment. Examples of supports include social support from family and friends and availability of information. Therefore, minimizing environmental barriers and building upon supports are promising intervention strategies for improving participation, especially for youth with disabilities, who might not yet have the necessary skills to manage environmental barriers to participation themselves [15]. Consequently, interventions that aim to improve participation via environmental modifications have emerged in the last decade. Examples include context-focused therapy for young children with cerebral palsy [16] and Teens Making Environment and Activity Modifications (TEAM) for youth with developmental disabilities [15]. The Pathways and Resources for Engagement and Participation (PREP) is another example of an environment-based participation-targeting intervention, which is designed for individuals across different ages and abilities [17] and employs a strength-based approach. This 12-week intervention provides youth and caregivers with one-on-one coaching to foster problem solving and self-advocacy skills in order to remove environmental barriers to, and build supports for participation [18].

In our recent study [19], PREP has been shown to improve the performance of youth with physical disabilities (n = 28) in three targeted, self-chosen, leisure community-based activities; yet, it has not been established whether this intervention could impact areas beyond its three targeted activities, resulting in overall changes in participation profile. Our prior research with a sub-sample of this cohort provides preliminary evidence of such an effect. Parents, through individual interviews (n = 12) [20], reported positive improvements in their child’s physical, emotional and social states following PREP. Another investigation of youth receiving PREP (n = 13) [21] indicated a shift in the types of activities performed, such as increased participation in social- and school-related activities, as measured using the Aday app, which is a 24 h activity log.

To complement these findings, the goal of this study is to systematically examine the effect of PREP on overall participation patterns using a standardized assessment. This was done by investigating a novel, distinct aspect of our existing dataset (n = 28) [19]. Specifically, our primary objectives were to examine the effect of PREP on (1) youths’ overall participation patterns in terms of frequency, involvement and desire for change in the home, school and community settings, and (2) the number of environmental barriers and supports to participation reported in these settings. A secondary objective was also set, exploring the association between baseline youth characteristics known to influence participation (in terms of physical functioning, motivation and family functioning) and rates of change of participation.

## 2. Materials and Methods 

### 2.1. Design 

A subsequent analysis of existing data generated by an Interrupted Time Series study previously conducted by Anaby et al. [19] was employed to detect overall changes in participation patterns following the 12-week PREP intervention. Specifically, participation patterns were examined at the first week of baseline (which lasted 4 weeks) as well as 4 weeks after the completion of the PREP intervention (12 weeks), resulting in a 20-week delay between pre- and post-points of time. The original dataset included 28 youths with mobility restrictions (i.e., due to cerebral palsy, spina bifida, musculoskeletal disorders), who were recruited from five major rehabilitation centers and two high schools in Greater Montreal, from both Anglophone and Francophone families. Youth who also had cognitive and/or communication impairments were included. Youth within the first-year post-severe brain injury, or within 4 months post orthopedic surgery, were excluded from the selection process, as their participation and functional levels may not have been stable. 

Of the 28 participants from the original study, a total of eight participants were excluded from the current study. Six participants were excluded because additional questionnaires (Dimensions of Mastery Questionnaire—DMQ, Activities Scale for Kids—ASK, Family Environment Scale—FES) were not administered to them at baseline. Two additional participants were excluded from the analysis as either pre- or post-intervention Participation and Environment Measure for Children and Youth (PEM-CY) data was incomplete. Thus, our current study included data from 20 participants. The included (n = 20) and excluded (n = 8) groups had an equal female-to-male ratio. The mean ages were similar (n = 20, mean = 14.4), (n = 8, mean = 15.13), and both groups had the same median age (median = 14.5). Mann–Whitney U tests indicated no significant differences between the groups in terms of number of health conditions (U = 74.5; *p* = 0.80258) and functional issues (U = 54.5; *p* = 0.20408). For further details on the original study design, see Anaby et al. [19].

### 2.2. Intervention 

The PREP is a 5-step intervention, i.e., (1) Make goals, (2) Map out a plan, (3) Make it happen, (4) Measure process and outcomes and (5) Move forward, aimed at improving participation in self-chosen activities by changing aspects of the environment and by engaging and coaching youth/parents. An occupational therapist met with each youth/family individually at their home/community where they jointly set three community-based participation goals that the youth aspired to engage in yet found difficult. Examples of desired activities included joining a sledge hockey team, taking cooking classes, going to the movies with friends and enjoying music in a social group, among others. A collaborative plan was then devised to identify and implement solution-based strategies for removing environmental barriers and leveraging existing supports. The therapist and family also built a “participation team” comprised of a range of stakeholders (i.e., family members, teachers, community instructors, volunteers, etc.) to assist in the execution of the plan. Further information about the intervention can be found in the PREP manual [17] and the online learning module [22]. 

To ensure treatment fidelity and adherence to PREP principles, all therapists (n = 6) completed a 6 h PREP training program. Ongoing expert consultation was also provided throughout the study. Additionally, intervention forms, completed by therapists, documenting strategies used in their interventions were reviewed. As expected, all intervention strategies illustrated modifications of the environment and none focused on changing the youth’s impairment. Effective environmental strategies included improving physical accessibility, adapting activity equipment, finding available programs, providing information about transportation, informing community agencies about how they could adapt their programs and provide accessible services and improving attitudes of others through education [19]. 

### 2.3. Procedure and Data Collection 

Informed consent and assent were obtained from parents and youth, approved by the Research Ethics Board of the Centre for Interdisciplinary Research in Rehabilitation of Greater Montreal. At baseline, during the first meeting with the therapist, several assessment measures were completed, including the Participation and Environment Measure—Children and Youth (PEM-CY) to measure participation patterns, Family Environment Scale (FES) to assess family functioning (contextual factors), Activities Scale for Kids (ASK) to measure physical functioning (activity limitation) and the Dimensions of Mastery Questionnaire (DMQ) to measure level of motivation (personal factors). The PEM-CY was also completed at follow-up (week 20).

### 2.4. Measures 

The PEM-CY is a parent-report measure that assesses participation frequency, involvement and desire for change across 25 sets of activities occurring in three different settings: home (10 activities), school (5 activities) and community (10 activities). Frequency is rated on an 8-point Likert scale (0 = never, 7 = daily) and level of involvement is ranked on a 5-point Likert scale (1 = minimally involved, 5 = very involved). Desire for change includes a 6-point nominal scale describing the type of change desired; however, for the purpose of this study it was treated dichotomously to indicate if a change in each activity was desired (yes/no). The PEM-CY also measures environmental factors, including barriers and supports to participation, within each of the three settings (12 items for home, 17 items for school and 16 items for community). The PEM-CY demonstrated moderate to good reliability (test re-test reliability, 0.58–0.95, internal consistency 0.59–0.91) as well as ability to distinguish between children with and without disabilities, supporting aspects of construct validity [23]. Factorial structure of participation frequency and involvement across all three settings was confirmed [11]. This measure has been used with children with Spina Bifida [24].

The FES is a valid and reliable self-report questionnaire used to assess the social environment of families [25]. It is composed of 90 self-report items that can be separated into 10 subscales. This study focused on two of the subscales of family functioning, Active–Recreation Orientation (i.e., family’s participation in social and recreational activities) and Intellectual–Cultural Orientation (i.e., family’s interest in political, intellectual and cultural activities), as there is evidence that these two subscales influence participation outcomes among children with physical disabilities [26]. For each subscale, a summary score was generated by converting true/false answers into a standardized score ranging from 0 to 100, where a score of 60 or more indicates that the subscale area is present to a high degree in the family. This measure has also been used with children with musculoskeletal disorders such as rheumatoid arthritis [27].

The DMQ is a parent-report tool used to measure a child’s self-perceived motivation. This measure contains 45 items, which assess the level of persistence to solve problems, mastery of tasks and the feelings associated with attempts of mastery. Parents indicate the degree that each item applies to their child using a five-point Likert scale. A general summary score ranging from 1 to 5 was generated, with scores of 5 indicating higher mastery motivation. This measure has been used with children with cerebral palsy [28], has adequate reliability and validity [29], and has been shown to be associated with children’s participation [30].

Aspects of activity limitation which are associated with participation [31] were measured using the ASK. The ASK is a valid and reliable self-report tool designed to measure physical functional issues for children and youth, experiencing activity limitations due to musculoskeletal disorders [32]. It includes 30 functional activities separated into 7 sub-domains (e.g., personal care and transfers) that rate independent performance of each activity using a 5-point scale. A summary score ranging from 0 to 100 is generated, where 0 indicates the greatest disability. A global rating of physical disability is also generated: mild (75 to 100), moderate (35 to 74) and severe (<35).

### 2.5. Data Analysis

#### 2.5.1. Primary Objective 1—Differences in Participation Levels in Each Setting following the PREP Intervention 

Setting-level and item-level mean scores of diversity (number of activities actually done), frequency (ranged from 0 to 7), involvement (ranged from 1 to 5), and number of activities in which parents wanted to see change were calculated pre- and post-PREP intervention.

Setting-level changes, i.e., changes in mean participation levels across an entire setting (home, school, community), pre- and post-intervention were analyzed using a paired t-test; this is based on the central limit theorem assumption that with a sample of 20 youth, the sampling distribution of the mean approximates a normal distribution. In cases where the number of responders was less than 20, a non-parametric test was used (Wilcoxon). Values of *p* < 0.05 were considered significant and 95% Confidence Intervals (CI) were calculated. Cohen’s *d* was used to estimate effect sizes, where *d* = 0.2 is considered a small effect, *d* = 0.5 is medium and *d* = 0.8 is large. SPSS Software Version 25 was used for all statistical calculations. Data was also analyzed descriptively to identify direction and amount of change.

Item-level mean scores were calculated for participation frequency and number of youths engaged in each of the activities, pre- and post-intervention. All item-level comparisons were graphed using radar plots to visually analyze the data. Items of activities were analyzed for significance when the amount of change was more likely to represent a change in one category/point within the frequency scale (e.g., from “once in a week” = 1 to “few times a week” = 2′). These values corresponded to a mean difference in frequency of greater than 0.5 points. Wilcoxon or paired t-tests were used depending on the number of responses per item. To reduce the number of item-level statistical comparisons, diversity scores (representing number of youths engaged in each activity) were only tested for statistical significance (using the Chi-square test) when a pre–post change was observed in at least 20% of the sample, an arbitrary set. Notably, item-level analyses were only performed for those setting-level mean scores which were statistically significant.

#### 2.5.2. Primary Objective 2—Differences in Environmental Barriers and Supports in Each Setting following the PREP Intervention 

Setting-level and Item-level scores for environmental barriers and supports were calculated pre- and post-intervention. Scores represent the number of parents (in percentages) who viewed the given environmental item as a barrier/support.

Setting-level mean scores, i.e., changes in the percentage of environmental barriers/supports reported in each setting (home, school, community) were analyzed using the same methods as objective 1: A paired t-test or a Wilcoxon test, as well as descriptively. 

Item-level mean scores were calculated for each of the environmental barriers/supports, pre- and post-intervention. Items in which a change of at least 20% of the sample was observed were statistically analyzed using Chi-square tests. All Item-level comparisons were displayed using radar plots, in terms of percentage of parents who considered the given environmental item to be a barrier/support. These radar plots were used to analyze data visually.

#### 2.5.3. Secondary Objective—Association between Youth’s Characteristics at Baseline and Rates of Change of Participation

To examine the secondary objective, exploratory analysis was done to investigate factors associated with change in participation scores that were found significant in objective 1. Exploratory variables considered were: youth functional levels (number of functional issues reported, ASK total score of physical functioning) motivation (i.e., DMQ gross motor persistence, mastery pleasure, negative reaction, object-oriented persistence, social persistence with children, social persistence with adults) and aspects of family functioning (FES active–recreation orientation scale standard score, FES intellectual-cultural orientation scale standard score). To identify patterns/association between change in participation and the explanatory variables, change scores (post-score − pre-score) were calculated and plotted against the baseline scores on the explanatory variables. A loess smoothed line (with span of 0.75) was added to each scatterplot to help identify any patterns visually.

## 3. Results

### 3.1. Participants 

Twenty youth (10 female) aged 12–18 years (mean = 14.4; standard deviation (SD) = 1.82) were included in this analysis. Up to seven health conditions were reported per youth (mean = 2.4, SD = 1.7; Interquartile range (IQR) 1 to 3), with the most common being orthopedic/movement impairments (70%), followed by speech/language impairments (50%), intellectual delay (25%) and vision impairment (25%). Number of functional issues ranged from 1 to 11 (mean = 5.1, SD = 3.01; IQR 3 to 7) including difficulty using hands to do activities (85%), moving around (72%), communicating with others (58%) and managing emotions (58%). The majority of the youth (68.8%) had a severe physical disability, as measured by the ASK. As shown in Table 1, levels of family functioning in terms of active–recreation and intellectual–cultural orientation were below 60, indicating a relativity low presence of these attributes. In terms of motivation, similar levels of mastery pleasure and gross motor persistence were observed, when compared to typically developing teens of a similar age [29]. The remaining domains of motivation approached normative levels, apart from negative reactions to failure. Further sociodemographic factors are also described in Table 1.

### 3.2. Differences in Participation and Environmental Scores in Each Setting

Following the PREP intervention, on average, youth participated significantly more often (*d* = 0.57, 95% CI [−0.79, −0.14]) and in greater ranges of activities (*d* = 0.51, 95% CI [−1.99, −0.51]) in the community setting with moderate effect sizes, and significantly less often in the home setting (*d* = 2.1, 95% CI [1.79, 2.96]), with a large effect size (Table 2). Youth also participated more often in school, yet a non-significant effect was observed. Levels of involvement and percentages of parents who desired change in activities remained relatively similar pre- and post-intervention across all settings. 

The results that follow are organized according to scale, restricted to those scales where a statistically significant change was observed.

### 3.3. Differences in Frequency Scores 

Following the PREP intervention a significant, moderate effect on participation was observed in the community setting (ES = 0.57, 95% CI [−0.79, −0.14]), where participation frequency increased, and a significant, large effect on participation was observed in the home setting (ES = 2.14, 95% CI [1.79, 2.96]), where participation frequency decreased. Additionally, a small, non-significant effect was observed in the school setting, indicating an increase in frequency levels (Table 2).

**Figure 1:**
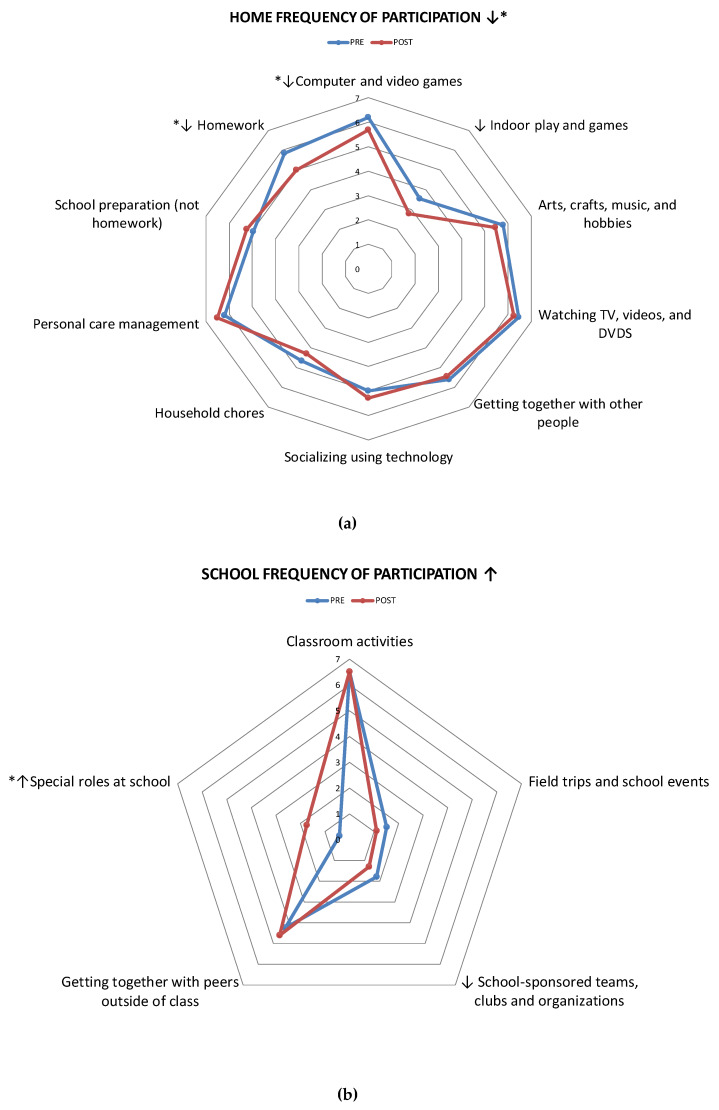

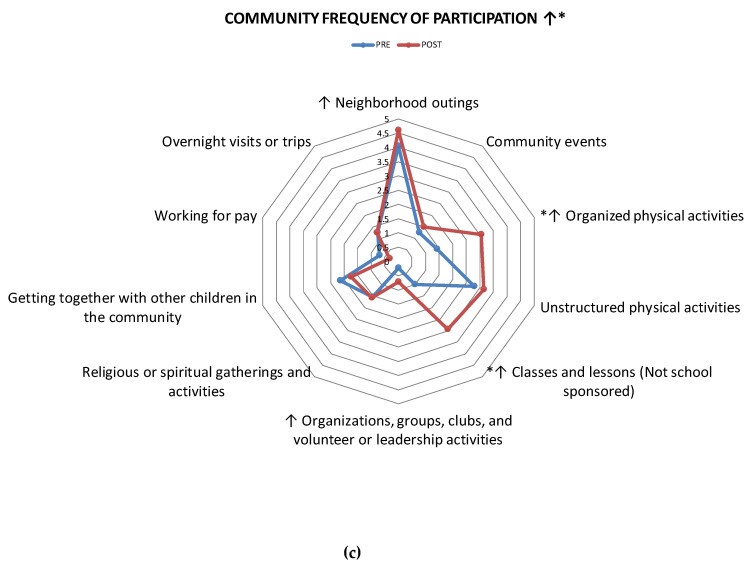
Frequency of participation in the home (**a**), school (**b**), and community settings (**c**) (n = 20). 0 = Never, 1 = Once in the last four months, 2 = Few times in the last four months, 3 = Once a month, 4 = Few times a month, 5 = Once a week, 6 = Few times a week, 7 = Daily. ↑/↓ = Mean increase/decrease of at least 0.5. * *p* < 0.05.

Changes in frequency at the Item-level (within each activity) indicated that across the three settings, nine activity sets out of 25 were found to have a pre-post difference equal to 0.5 or greater, five of which illustrated a statistically significant change (Figure 1). Children participated significantly less at home, specifically in *computer and video games* (Z = −2.33, *p* = 0.02), and *homework* (Z = −2.043, *p* = 0.041). They significantly took on more *special roles at school* (t = −2.46, *p* = 0.024) such as lunchroom supervisor or student mentor roles, among others. In the community setting, youth significantly participated more often in two activity sets: *organized physical activities* (t = −3.11, *p* = 0.006) and *classes and lessons* (t = −2.614, *p* = 0.018), and a positive non-significant change was observed in *organizations, groups, clubs, and volunteer or leadership activities* and *neighborhood outings.*

### 3.4. Differences in Diversity Scores 

As previously mentioned, a significant moderate effect on participation diversity was observed in the community section (*d* = 0.51, 95% CI [−1.99, −0.51]), where youth took part in a greater number of activities following the intervention. The diversity scores of home and school activities remained similar post-intervention (Table 2).

**Figure 2:**
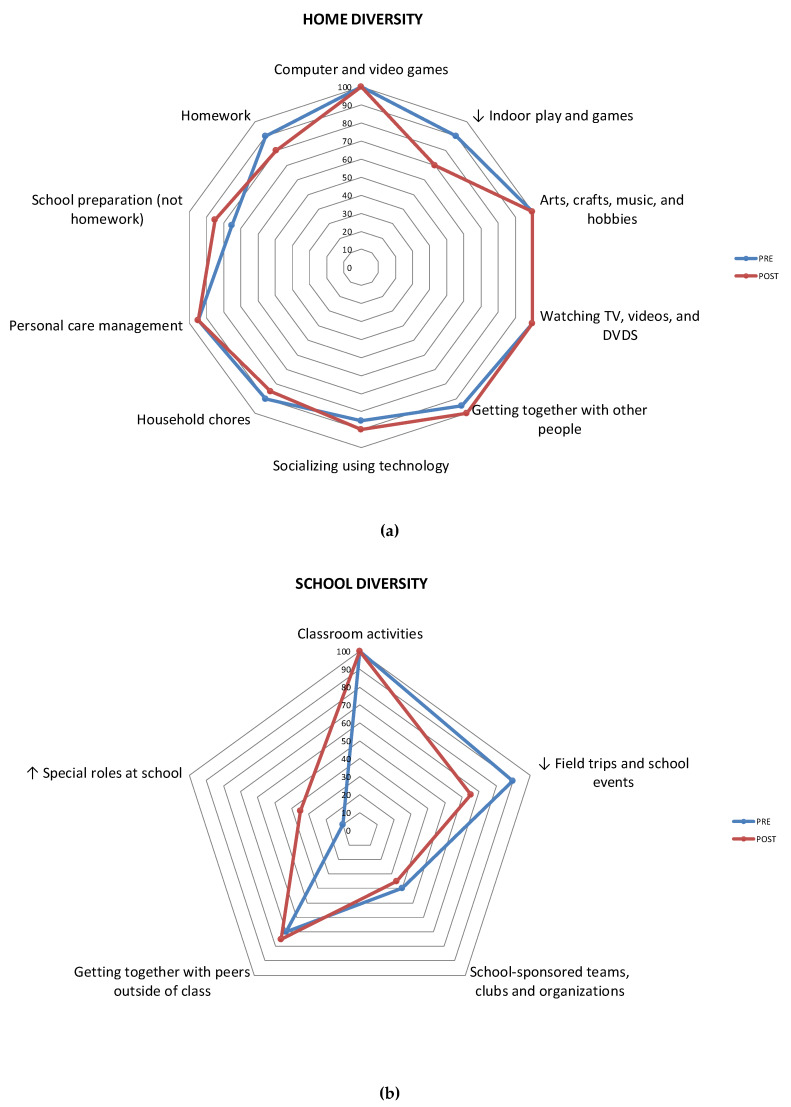

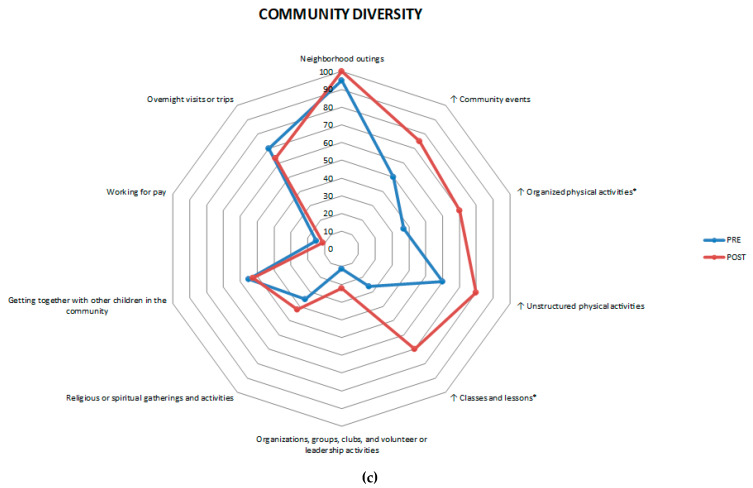
Percentage of youth (n = 18 to 20) participating in each activity in the home (**a**), school (**b**), and community (**c**) settings. ↑/↓ = Increase/decrease in at least 20% of sample. * *p* < 0.05.

Looking at the item-level scores, across the three settings, there were seven activity types out of 25 in which a change of 20% of the sample occurred, two of which were statistically significant based on Chi-square tests. Specifically, in the community, there were more youth participating in *organized physical activities* (χ^2^ = 4.31, *p* = 0.037) and *classes/lessons* (χ^2^ = 7.44, *p* = 0.006). Specific trends (non-significant) were also observed in all three settings. In the home, fewer youth participated in *indoor play and games*. In the school, fewer youth attended *field trips and school events*, and more youth took on *special roles at school*. In the community, there were more youth participating in *community events* and *unstructured physical activities* (Figure 2). 

### 3.5. Differences in Environmental Barriers 

**Figure 3:**
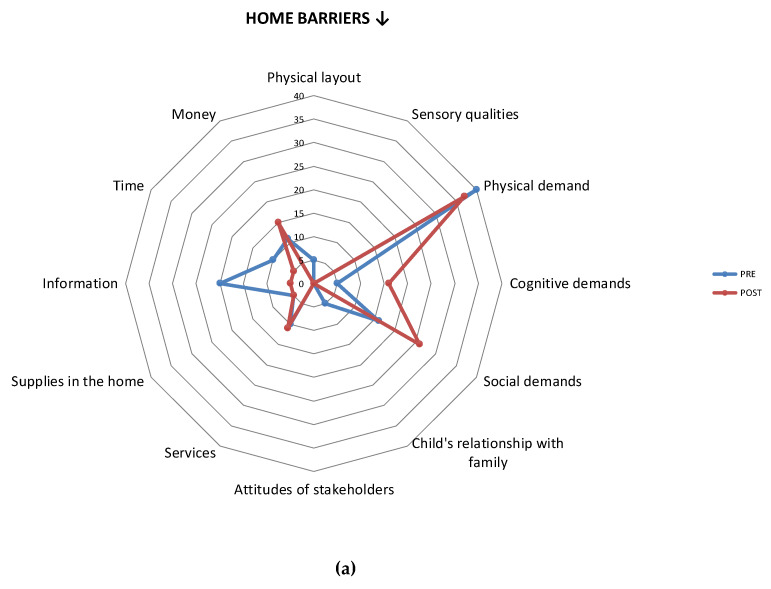

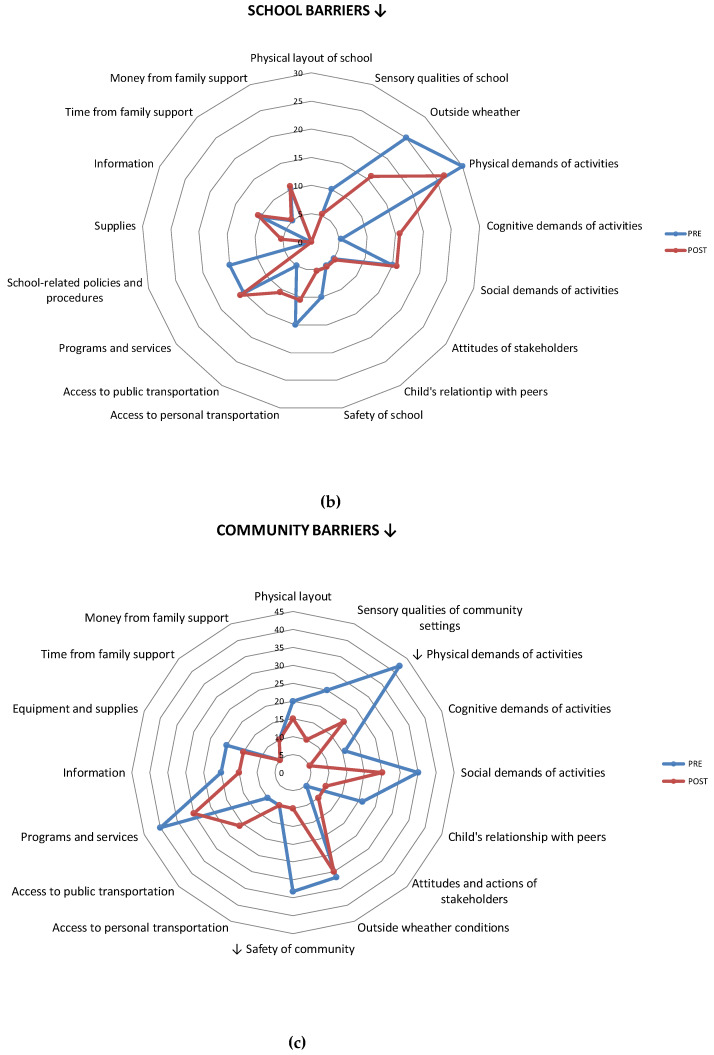
Percentage of parents (out of 20) who reported an environmental component as being a barrier in the home (**a**), school (**b**), and community (**c**) settings. ↓ = Decrease in at least 20% of sample. * *p* < 0.05.

While the mean number of setting-level barriers did not change significantly after PREP in any of the settings (Table 2), Item-level examination revealed a change in a range of barriers across all settings. In the community, parents reported a reduction in most barriers (11/16), with 20% fewer parents viewing *physical demands of activities*, and 25% fewer parents viewing *safety of the community* as barriers. Interestingly, a few specific environmental barriers in the home and school slightly increased following PREP, particularly those related to the cognitive and social demands of the activity (Figure 3).

### 3.6. Differences in Environmental Supports

**Figure 4:**
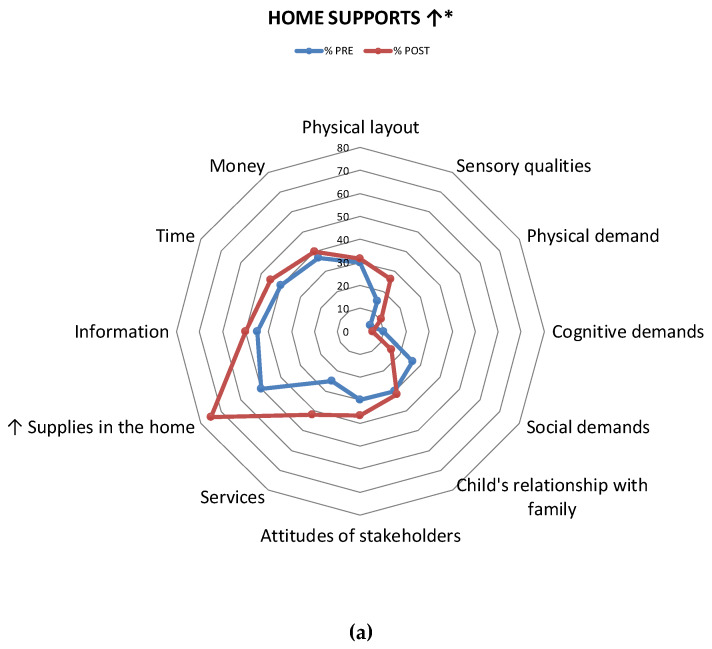

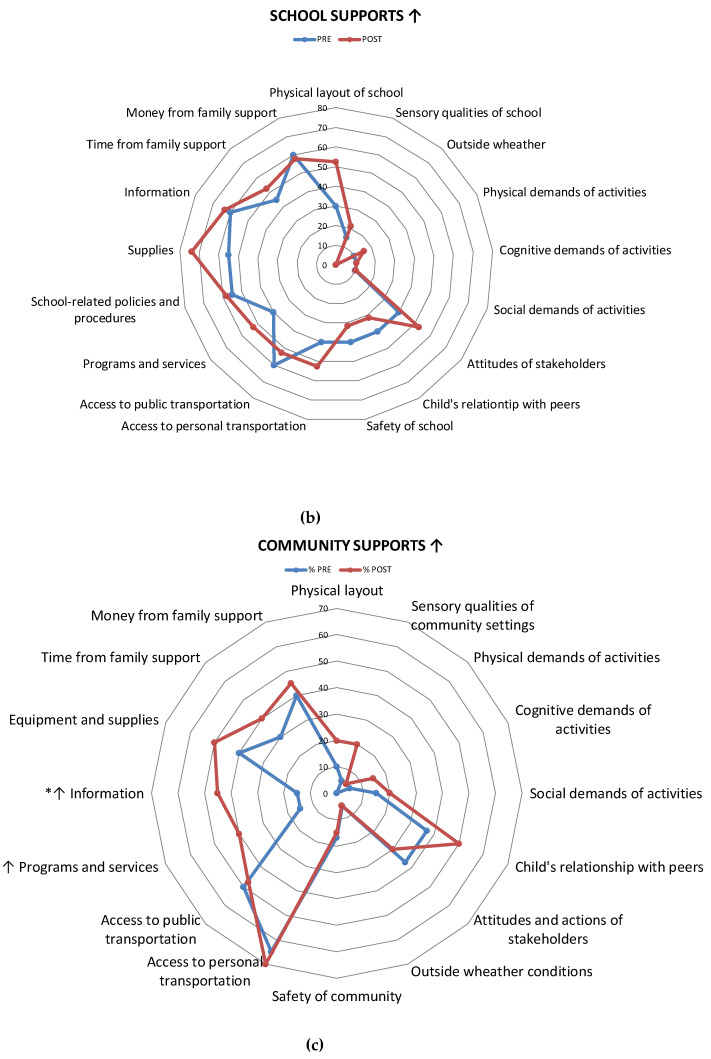
Percentage of parents (out of 20) who consider an environmental component as being a source of support in the home (**a**), school (**b**), and community (**c**) settings. ↑ = Increase in at least 20% of sample. * *p* < 0.05.

As shown in Table 2, there was a non-significant increase in the mean number of supports in the home after PREP, with a small effect size (*d* = 0.24, 95% CI [−15.77, 5.77]). Mean number of supports remained fairly similar in the school and increased in the community with median effect size (*d* = 0.67, 95% CI [−17.20, 0.33]) approaching statistical significance (*p* = 0.058). Item-level comparisons indicated that parents reported an initial trend of increase in 8/12 supports in the home, 10/17 in the school and 12/16 in the community. Across all three settings, 20% of parents or more added a support in four environmental supports, one of which was statistically significant, i.e., availability of information (15% of parents pre versus 45% post, χ^2^ = 4.28, *p* = 0.038; Figure 4). Overall, more parents viewed *supplies* in the home (e.g., sports equipment, craft supplies), *physical layout* of the school, availability of community *programs* and community *information* as supports after PREP.

### 3.7. Secondary Objective—Association between Child’s Characteristics at Baseline and Changes in Participation 

Secondary objective analysis was performed on the three scores found to have statistically significant pre–post differences in objective 1 (i.e., home participation frequency, community participation frequency, and diversity). Visual examination of scatterplots indicated that none of the youth’s characteristics at baseline were associated with rate of change in participation scores, with the exception of level of physical disability, measured by the ASK, where initial trends of association were observed. Specifically, youth with more severe disabilities tend to change slightly more in their participation frequency in the home setting, whereas in the community, changes to their participation appear less evident. Family functioning and youth motivation at baseline did not seem to influence change in participation patterns following PREP.

## 4. Discussion

### 4.1. Changes in Activities and Settings 

After PREP, youth participation frequency significantly increased in the community setting, while it decreased in the home setting. This shift in participation patterns, supported by moderate to large effect sizes, is encouraging and positive as previous research shows that youth with physical disabilities tend to spend more time alone and at home [8]. Moreover, a change towards more community ‘out-of-home’ activities done with others is considered beneficial. In general, the majority of observed changes occurred in the community, which further supports the impact demonstrated by PREP in previous studies [19,20,21], and reflects the area in which the targeted activities took place (i.e., the community). The positive changes in specific activities within other settings such as the school (i.e., taking on special roles in school), found in this study, may indicate that youth and parents were applying skills they had gained during PREP in order to explore new opportunities in additional environments. This finding coincides with a qualitative study [20], in which parents whose child received the intervention, indicated that youth “had gained tools” to apply to other settings. Specifically, they expressed interest and showed initiative in taking on new roles and activities in school, for instance, an environment that was not directly targeted by PREP [20]. 

Youth also demonstrated changes in the types of activities that they were participating in. Participation frequency in sedentary leisure activities at home, such as *computer and video games* decreased, while frequency in active forms of leisure or social leisure in the community, such as *organized physical activities* and *classes and lessons* increased. These changes confirm a trend regarding decreased frequency of participation in digital media activities following PREP, which was initially observed in a previous study of a sub-sample of this cohort [21]. Overall, this initial shift in the types of activities undertaken supports patterns of participation and active lifestyle behaviors that are health-promoting. 

Following PREP, involvement levels remained stable. This may be due, in part, to the length of study. It is possible that a 12-week period of time was not sufficient for the youth to experience the level of comfort and sense of social inclusion and belonging that comes with familiarity (of the new activity) often necessary to become fully involved [33]. As such, implementing additional prolonged follow-ups may allow changes to be observed in involvement scores, illustrating the subjective experience that is derived from the activity. Given that the PEM-CY was not completed by the youth themselves, it is possible that subtle changes in level of involvement, a highly subjective construct, would have been difficult to detect by a proxy. Regarding desire for change, it is difficult to determine whether changes occurred or not without qualitative data to complement interpretation. For example, an increase in the number of activities in which change is desired could indicate a newfound motivation, as PREP may have provided parents and youth with new insight into their participation capacity. Alternatively, an increase in activities parents wish to see change in, may indicate that parents and youth are less satisfied with the current level of participation. In-depth interviews, where participants could reflect on their PEM-CY results and the cause of changes observed, could complement interpretation. 

### 4.2. Skill Implementation 

Families likely implemented skills and knowledge obtained through PREP to modify their environments, as shown by the descriptive changes in certain barriers and supports across all settings. For example, the decrease observed in *physical demands* of activities as a barrier in the community setting likely results from coaching families (and other stakeholders) on ways of grading and adapting specific activities to youth’s abilities, making activities more accessible and manageable. In addition, accessibility to resources, such as *supplies* in the home and *information* in the community (about activities, services and programs), were perceived as supports by more parents following PREP. This may reflect families gaining new knowledge about the resources available to them and new connections to other families with children with disabilities, allowing for the exchange of information, as well as developing more advanced advocacy skills. This is a valuable finding as parents who are equipped with knowledge and skills to improve their child’s participation, often become “knowledge brokers”, who confidently explore opportunities for their families [14]. 

As expected, environmental barriers encountered in the community displayed a pattern that suggests a post-intervention decrease. However, a few specific barriers were encountered more often in the home and school after PREP, especially those barriers related to the demands of the activity. These specific barriers may have been reported due to novel challenges encountered while starting new activities, such as *cognitive demands*. Additionally, at baseline, parents may not have considered that certain factors could act as barriers. Such an effect has also been reported by Kramer et al. [34], where parents identified significantly more barriers after applying a structured problem-identification strategy. It is plausible that the more one participates, the more barriers one encounters. Further studies are needed to examine this assumption. 

### 4.3. The Impact of Child and Family Characteristics on Rates of Change

None of the children’s characteristics measured at baseline were associated with rates of changes in PEM-CY scores that were found significant. This may suggest that the PREP intervention was beneficial to various youth and families regardless of their level of motivation and family functioning. Presumably, this speaks to the nature of the intervention where youth participate in an activity of choice (which can increase motivation) and where family barriers are addressed (as environmental barriers to remove). Physical functioning at baseline showed an initial trend of association with changes in participation outcomes which concur with previous research, where the effects of PREP were influenced by the number of functional issues at baseline [19]. This may be explained by the fact that PREP considers aspects of motivation and family environment but does not directly target functional issues. However, given the sample size, there was not enough power to detect clear patterns of association between child/family factors and changes in participation. 

### 4.4. Limitations, Strengths and Future Directions 

While this study included a relatively small sample size, we had sufficient power to detect changes in participation (primary objective), which has contributed an additional piece of evidence towards PREP’s effectiveness as well as preliminary evidence towards its ability to foster positive change in participation beyond its three specific targeted activities. In addition, this was the first study to evaluate pre-post data using the PEM-CY, providing support for the potential ability of this tool to detect change following an intervention. However, as the PEM-CY is a parent-report measure, it may not have captured the youths’ subjective experience, particularly in the desire for change and involvement scales, as those aspects of participation did not display significant differences post-intervention. Furthermore, the lack of qualitative information may have limited the interpretability of changes in these areas of participation. Overall, the results from this study are promising and warrant larger and prolonged trials in order to better capture all potential changes resulting from the PREP approach. In addition, combining results with qualitative interviews would better support interpretation of the PEM-CY, particularly with regards to parent’s desire for change. Finally, further studies could also contribute evidence towards the PEM-CYs responsiveness to change.

## 5. Conclusions

This study contributes to a growing body of evidence that environmental-based interventions, such as PREP, are effective at enhancing participation. By equipping families with solution-based strategies, PREP may empower them to explore new opportunities beyond their initial target goals and potentially carry-over skills into other areas of participation. Further, larger and prolonged studies can be used to capture change in the subjective aspects of participation (i.e., involvement and desire for change). Consequently, this can support the multiple benefits that can be generated by one single intervention, improving the provision of rehabilitation services in pediatrics. 

## Figures and Tables

**Table 1:** Sample Characteristics (n = 20).

Variable	n	%
**Class type**		
Regular classroom	3	15
Both regular and special classroom	3	15
Special education class	13	65
Other (International program)	1	5
**Community type**		
Major urban	7	35
Suburban	7	35
Small town	2	10
Missing	4	20
**Language**		
English	2	10
French	7	35
Other (Spanish and Arabic)	2	10
Bilingual (English or French with Bulgarian, Arabic, Portuguese, Hebrew, or Creole)	9	45
**Age of family member**		
30–39	3	15
40–49	13	65
50–59	4	20
**Education level of family member**		
High school or less	3	15
Some college /university or technical training 1-year min	4	20
Graduate college /university	11	55
High school or less	2	10
	**Mean**	**SD**
**Family Environment Scale (FES)**		
Active-Recreation Orientation (n = 20)	48.85	11.86
Intellectual-Cultural Orientation (n = 20)	52.50	8.44
**Dimensions of Mastery Questionnaire (DMQ)**		
Persistence at object or cognitive tasks (n = 18)	3.57	0.82
Gross motor persistence (n = 19)	2.99	0.76
Social mastery motivation with adults (n = 20)	3.47	0.80
Social mastery motivation with peers/children (n = 18)	3.24	0.93
Mastery pleasure (n = 18)	3.90	0.73
Negative reactions in mastery situations (n = 19)	3.03	0.92
**Activities Scale for Kids (ASK)**		
ASK total score (n = 19)	mild	15.9%
	moderate	15.9%
	severe	68.8%

**Table 2:** Setting-level Participation and Environment Measure for Children and Youth mean scores in the home, school, and community (n = 20).

	PEM-CY Scale (Range/Unit)	Pre Scores Mean (SD)	Post Scores Mean (SD)	95% CI	t	ES
**Home**	Frequency (0–7)	5.43 (1.11)	3.05 (1.02)	1.79, 2.96	8.456 ***	2.144
	Diversity (0–10)	9.20 (1.24)	9.00 (1.45)	−0.13, 0.52	1.285	0.16
	Level of involvement (1–5)	4.06 (0.48)	3.97 (0.82)	−0.22, 0.39	0.586	0.17
	Number of activities desired for change (0–10)	5.00 (2.38)	5.30 (2.54)	−1.39, 0.785	−0.578	0.12
	Number of environmental barriers (in %)	10.42 (14.02)	9.58 (11.24)	−4.05, 5.72	0.357	0.06
	Number of environmental supports (in %)	28.33 (20.84)	33.33 (17.10)	−15.77, 5.77	−0.972	0.24
**School**	Frequency (0–7)	2.84 (1.13)	3.05 (1.02)	−0.79, 0.36	−0.782	0.189
	Diversity (0 –5)	3.05 (0.94)	3.10 (1.25)	−0.44, 0.34	−0.271	0.052
	Level of involvement (1–5)	3.70 (1.08)	3.55 (1.17)	−0.40, 0.70	0.571	0.14
	Number of activities desired for change (0–5)	2.95 (1.57)	2.70 (1.59)	−0.49, 0.99	0.705	0.16
	Number of environmental barriers (in %)	10.59 (9.27)	9.12 (8.20)	−3.07, 6.01	0.677	0.16
	Number of environmental supports (in %)	35.88 (18.39)	38.53 (19.88)	−13.12, 7.83	−0.529	0.14
**Community**	Frequency of participation (0–7)	1.68 (0.91)	2.15 (0.78)	−0.79, −0.14	−3.017 **	0.57
	Diversity (score 0–10)	4.50 (2.44)	5.75 (1.83)	−1.99, −0.51	−3.526 **	0.51
	Level of involvement (1–5)	3.73 (1.15)	3.97 (0.82)	−0.82, 0.33	−0.894	0.17
	Number of activities desired for change (0–10)	6.8 (2.28)	6.05 (2.96)	−0.74, 2.24	1.06	0.33
	Number of environmental barriers (in %)	17.50 (16.30)	15.00 (10.42)	−4.30, 9.30	0.769	0.15
	Number of environmental supports (in %)	23.75 (13.23)	32.19 (19.37)	−17.20, 0.33	−2.015 ^a^	0.67

** *p* < 0.01; *** *p* < 0.001; ^a^ = 0.058; ES = Effect Size represented by Cohen’s *d.*

## References

[1] WHO (2001). International Classification of Functioning, Disability and Health.

[2] Heah T., Case T., McGuire B., Law M. (2007). Successful participation: The lived experience among children with disabilities. Can. J. Occup. Ther..

[3] Larson R.W. (2000). Toward a psychology of positive youth development. Am. Psychol..

[4] Law M. (2002). Participation in the occupations of everyday life. Am. J. Occup. Ther..

[5] Imms C., Mathews S., Richmond K.N., Law M., Ullenhag A. (2016). Optimising leisure participation: A pilot intervention study for adolescents with physical impairments. Disabil. Rehabil..

[6] Jarus T., Anaby D., Bart O., Engel-Yeger B., Law M. (2010). Childhood participation in after-school activities: What is to be expected?. Br. J. Occup. Ther..

[7] Gorter J.W., Stewart D., Woodbury-Smith M. (2011). Youth in transition: Care, health and development. Child Care Health Dev..

[8] Engel-Yeger B., Jarus T., Anaby D., Law M. (2009). Differences in patterns of participation between youths with cerebral palsy and typically developing peers. Am. J. Occup. Ther..

[9] Michelsen S.I., Flachs E.M., Damsgaard M.T., Parkes J., Parkinson K., Rapp M., Arnaud C., Nystrand M., Colver A., Fauconnier J. (2014). European study of frequency of participation of adolescents with and without cerebral palsy. Eur. J. Paediatr. Neurol. EJPN Off. J. Eur. Paediatr. Neurol. Soci..

[10] Bedell G., Coster W., Law M., Liljenquist K., Kao Y.C., Teplicky R., Anaby D., Khetani M.A. (2013). Community participation, supports, and barriers of school-age children with and without disabilities. Arch. Phys. Med. Rehabil..

[11] Anaby D., Law M., Coster W., Bedell G., Khetani M., Avery L., Teplicky R. (2014). The mediating role of the environment in explaining participation of children and youth with and without disabilities across home, school, and community. Arch. Phys. Med. Rehabil..

[12] Krieger B.A.-O., Piškur B., Schulze C., Jakobs U., Beurskens A., Moser A. (2018). Supporting and hindering environments for participation of adolescents diagnosed with autism spectrum disorder: A scoping review. PLoS ONE.

[13] Anaby D., Hand C., Bradley L., Direzze B., Forhan M., Digiacomo A., Law M. (2013). The effect of the environment on participation of children and youth with disabilities: A scoping review. Disabil. Rehabil..

[14] Willis C., Girdler S., Thompson M., Rosenberg M., Reid S., Elliott C. (2017). Elements contributing to meaningful participation for children and youth with disabilities: A scoping review. Disabil. Rehabil..

[15] Kramer J.M., Roemer K., Liljenquist K., Shin J., Hart S. (2014). Formative evaluation of project TEAM (teens making environment and activity modifications). Intell. Dev. Disabil..

[16] Law M., Darrah J., Pollock N., Wilson B., Russell D.J., Walter S.D., Rosenbaum P., Galuppi B. (2011). Focus on function: A cluster, randomized controlled trial comparing child- versus context-focused intervention for young children with cerebral palsy. Dev. Med. Child Neurol..

[17] Law M., Anaby D., Teplicky R., Turner L. Pathways and Resources for Engagement and Participation (PREP): A Practice Model for Occupational Therapists. https://www.canchild.ca/en/shop/25-prep.

[18] Law M., Anaby D., Imms C., Teplicky R., Turner L. (2015). Improving the participation of youth with physical disabilities in community activities: An interrupted time series design. Aust. Occup. Ther. J..

[19] Anaby D., Law M., Feldman D., Majnemer A., Avery L. (2018). The effectiveness of the Pathways and Resources for Engagement and Participation (PREP) intervention: Improving participation of adolescents with physical disabilities. Dev. Med. Child Neurol..

[20] Anaby D., Mercerat C., Tremblay S. (2017). Enhancing youth participation using the PREP intervention: Parents’ perspectives. Int. J. Environ. Res. Public Health.

[21] Anaby D., Vrotsou K., Kroksmark U., Ellegard K. (2019). Changes in participation patterns of youth with physical disabilities following the Pathways and Resources for Engagement and Participation intervention: A time-geography approach. Scand. J. Occup. Ther..

[22] Anaby D., Law M., Teplicky R., Turner L. PREP—Pathways and Resources for Engagement and Participation. https://www.prepintervention.ca/.

[23] Coster W., Bedell G., Law M., Khetani M.A., Teplicky R., Liljenquist K., Gleason K., Kao Y.-C. (2011). Psychometric evaluation of the participation and environment measure for children and youth. Dev. Med. Child Neurol..

[24] Bakaniene I., Prasauskiene A. (2019). Patterns and predictors of participation in children and adolescents with spina bifida. Disabil. Rehabil..

[25] Moos R.H., Moos B.S. (2009). Family Environment Scale Manual.

[26] King G., Law M., Hanna S., King S., Hurley P., Rosenbaum P., Kertoy M., Petrenchik T. (2006). Predictors of the leisure and recreation participation of children with physical disabilities: A structural equation modeling analysis. Child. Health Care.

[27] Gerhardt C.A., Vannatta K., McKellop J.M., Zeller M., Taylor J., Passo M., Noll R.B. (2003). Comparing parental distress, family functioning, and the role of social support for caregivers with and without a child with juvenile rheumatoid arthritis. J. Pediatr. Psychol..

[28] Majnemer A., Shevell M., Law M., Poulin C., Rosenbaum P. (2010). Level of motivation in mastering challenging tasks in children with cerebral palsy. Dev. Med. Child Neurol..

[29] Morgan G.A., Leech N.L., Barrett K.C., Busch-Rossnagel N.A., Harmon R.J. (2000). The Dimensions of Mastery Questionnaire.

[30] Majnemer A., Shevell M., Law M., Birnbaum R., Chilingaryan G., Rosenbaum P., Poulin C. (2008). Participation and enjoyment of leisure activities in school-aged children with cerebral palsy. Dev. Med. Child Neurol..

[31] Beckung E., Hagberg G. (2002). Neuroimpairments, activity limitations, and participation restrictions in children with cerebral palsy. Dev. Med. Child Neurol..

[32] Young N.L., Williams J.I., Yoshida K.K., Wright J.G. (2000). Measurement properties of the activities scale for kids. J. Clin. Epidemiol..

[33] Palisano R.J., Begnoche D.M., Chiarello L.A., Bartlett D.J., McCoy S.W., Chang H.-J. (2012). Amount and Focus of physical therapy and occupational therapy for young children with cerebral palsy. Phys. Occup. Ther. Pediatr..

[34] Kramer J.M., Helfrich C., Levin M., Hwang I.T., Samuel P.S., Carrellas A., Schwartz A.E., Goeva A., Kolaczyk E.D. (2018). Initial evaluation of the effects of an environmental-focused problem-solving intervention for transition-age young people with developmental disabilities: Project TEAM. Dev. Med. Child Neurol..

